# Antimicrobial Resistance and Virulence Determinants of *Escherichia coli* Isolates from Raw Milk of Dairy Cows with Subclinical Mastitis

**DOI:** 10.3390/ani15131980

**Published:** 2025-07-05

**Authors:** Ntelekwane George Khasapane, Olga de Smidt, Kgaugelo Edward Lekota, Jane Nkhebenyane, Oriel Thekisoe, Tsepo Ramatla

**Affiliations:** 1 Centre for Applied Food Safety and Biotechnology, Department of Life Sciences, Central University of Technology, 1 Park Road, Bloemfontein 9300, South Africa; olga@cut.ac.za (O.d.S.); sknhebenn@cut.ac.za (J.N.); tramatlaa@cut.ac.za (T.R.); 2 Unit for Environmental Sciences and Management, North-West University, Potchefstroom 2531, South Africa; lekota@nwu.ac.za (K.E.L.); thekisoeo@nwu.ac.za (O.T.)

**Keywords:** Shiga toxin-producing *Escherichia coli*, subclinical mastitis, MALDI-TOF MS, PCR, antimicrobial resistance

## Abstract

Subclinical mastitis (SCM) is a stealthy but devastating challenge in the dairy industry, leading to economic losses and hindering efforts to achieve milk self-sufficiency. This study investigated the prevalence of SCM, antimicrobial resistance, and virulence profiles of *Escherichia coli*. A total of 174 milk samples were analyzed using the California mastitis test (CMT), somatic cell counts (SCCs), bacteriological culture, MALDI-TOF MS, and polymerase chain reaction (PCR). The findings revealed that the SCM prevalence was 68/174 (39.08%) based on CMT and SCC. Among SCM-positive samples, 60/68 (88.23%) were identified as *E. coli,* confirmed by MALDI-TOF MS and PCR assay. The most frequently detected serogroups were 0113 (11.6%) and 0113 (3.3%). Additionally, the genes for *Stx1* and *Stx2* were also detected in nine (15%) and one (1.7%), respectively. Antimicrobial susceptibility tests showed widespread resistance, with *E. coli* isolates demonstrating resistance to penicillin in 43 (71.6%), followed by ciprofloxacin in 42 (70%) and gentamicin in 18 (30%). A larger proportion of the *E. coli* strains (100%) harbored the *bla*_VIM_ gene, while 23 (38.3%), 20%, 20%, and 1.47% contained *bla*_KPC_, *bla*_NMD_, *suli1*, and *msrA*. Thirty (50%) isolates were considered multidrug-resistant (MDR). These findings underscore the urgent need for enhanced surveillance and antibiotic stewardship in dairy farming. The presence of MDR *E. coli* in SCM poses a dual threat of potential transmission to humans and treatment failures in mastitis management. This study highlights the importance of proactive control strategies to mitigate the spread of antimicrobial resistance in livestock and beyond.

## Simple Summary

Subclinical mastitis (SCM) is a major but often undetected issue in dairy farming, causing economic losses and affecting milk production. This study analyzed 174 milk samples and found SCM in 39.08% of them. Of these SCM-positive cases, 88.23% were caused by *Escherichia coli*. The most common serogroups detected were O113 and O111, and virulence genes *Stx1* and *Stx2* were found in 15% and 1.7% of isolates, respectively. Alarmingly, *E. coli* showed high resistance to several antibiotics, particularly penicillin (71.6%), ciprofloxacin (70%), and gentamicin (30%). All isolates carried the *blaVIM* resistance gene, and many also harbored *blaKPC*, *blaNDM*, *sul1*, and *msrA*. Half of the *E. coli* isolates were multidrug-resistant (MDR). These results highlight the critical need for better monitoring and responsible antibiotic use in dairy farming to prevent the spread of resistant bacteria to humans and improve mastitis treatment outcomes.

## 1. Introduction

Mastitis in cattle is one of the most significant illnesses that causes substantial financial losses in animal farms in developing nations and most of the world’s nations [1]. The expulsion of sick animals from the herd, significant milk production deficiencies, and high veterinary medication expenses are all consequences of mastitis [2]. Furthermore, a careful zoonotic viewpoint links mastitis to separating different bacteria and their harmful compounds in milk [3]. The ability of disease-causing microbes and their toxins to permeate the food chain and cause major foodborne illnesses has made mastitis a clear health risk [4], as noted by Oliver et al. [5] and Hennekinne et al. [6]. A significant cause of symptomatic and asymptomatic mastitis in dairy cows is the bacterium *E. coli*, although many other microbes can also lead to this condition [7].

Most *E. coli* strains are harmless, but some can cause foodborne diseases, such as those that generate Shiga toxins [8]. Humans are mostly exposed to this pathogen by consuming contaminated food, such as unpasteurized milk and dairy products [9]. While Shiga toxins (*Stx1* and *Stx2*) and intimin (*eae*) are among the numerous extremely pathogenic genes produced by *E. coli*, they are thought to be the most frequently found genes in cows with mastitis symptoms, which poses a clear risk to human health. Through direct contact with diseased animals or the consumption of food, water, or vegetables contaminated with animal feces, humans can occasionally be infected with STEC, which first appeared as a foodborne pathogen. STEC infections can thus be spread from person to person with ease. STEC isolates, particularly serotype O157:H7, can cause a variety of illnesses, from mild diarrhea to hemolytic uremic syndrome (HUS) and hemorrhagic colitis (HC). Children, the elderly, and patients with impaired immune systems are usually affected by these problems [9]. Naturally occurring after gastrointestinal infections with STEC, the systemic sickness of HC usually starts with diarrhea and cramping in the abdomen, followed by bloody diarrhea. Furthermore, in nearly 80% of cases in both children and adults, HUS, which is defined by acute renal failure, changes from bloody diarrhea at lunchtime to bloody diarrhea after one to five days [10]. Worldwide, the emergence of multidrug-resistant *E. coli* strains from clinical samples and mastitis milk is considered a public health concern [11]. The remarkable evolution of multidrug-resistant *E. coli* strains indicates that the strains from various animals and those from human clinical samples are closely correlated, according to earlier scientific research [12].

Antibiotic resistance to different pathogens is a vital community health issue that has been linked to increasing infection rates in some parts of the world [13]. According to Frieri et al. [14], multidrug-resistant bacteria are difficult to treat and may not even be curable with traditional antimicrobial medications. According to the World Health Organization, one of the greatest threats to public health in the twenty-first century is the resistance of different microorganisms to numerous antibiotics. This worldwide issue has compelled the hunt for new drugs with lower resistance.

The susceptibility of *E. coli* to various antibiotics has been found to decline recently. *E. coli,* which produces extended-spectrum *β*-lactamase (ESBL), is expanding globally in humans and farm animals. ESBL-encoding genes on mobile elements, the transfer of ESBL gene-carrying plasmids, and high-virulence gene content are some of the mechanisms linked to its successful dissemination [15]. According to Yu et al. [16], food animals and animal products contain a range of tetracycline resistance genes (TRGs) and aminoglycoside-modifying enzyme (AME) genes. Currently, there is limited public health surveillance data on the prevalence of *E. coli* in the research area. In such a context, it is challenging to define events and directions of transmission and evaluate the risk of disease transmission between humans, cattle, and the environment [17]. The aim of this study was therefore to characterize the molecular and phenotypic characteristics of *E. coli* strains isolated from the milk of dairy cows with subclinical mastitis from the Free State in South Africa.

## 2. Materials and Methods

### 2.1. Sampling and Mastitis Screening

In total, 174 milk samples were collected from dairy cows with subclinical mastitis on six farms distributed in the Thabo Mofutsanyana District Municipality of Free State Province in March 2024 (Supplementary Figure S1). To avoid cross-contamination, each cow’s udder was cleaned with distilled water and dried with a disposable paper towel before sample collection. A 10 mL sample of milk was collected from each cow after the teats were cleaned using towels and disinfected with 70% ethanol. A cow was defined as having subclinical mastitis after being subjected to screening for SCM using a somatic cell count (SCC) assay from Mérieux NutriSciences(South Africa, Cape Town). The SCC results were scored and interpreted as a healthy quarter if the SCCs were ≤100,000 cells/mL milk; weakly positive quarter if the SCCs were between >100,000 <500,000 cells/mL milk; distinctly positive if the SCCs were >500,000 <1,000,000 cells/mL milk; and strongly positive if the SCCs were ≥1,000,000 cells/mL milk, as recommended by Karzis et al. [18] and Khasapane et al. [19].

### 2.2. Bacterial Isolation and Identification

Milk samples (10 μL) were streaked onto Harlequin^®^ *E. coli*/Coliform Agar (Neogen, Scotland, UK) and incubated for 24 to 48 h at 37 °C. The sample culture was deemed negative if no bacterium could be isolated following the direct plating process. On the other hand, samples that isolated more than two bacterial species were deemed tainted. Colonies with the same chromogenic characteristics (blue-green colonies) were reisolated on nutrient agar (Neogen, Scotland, UK), and single colonies were sequentially transferred to create a pure culture. Then, as previously mentioned by Cameron et al. [20], the isolates were identified using matrix-assisted laser desorption ionization–time-of-flight (MALDI-TOF) mass spectrometry using MALDI-Biotyper 3.0 software (Bruker Daltonics, Bremen, Germany). Each identification of an isolate was performed twice. If isolates were not resolved after two rounds of MALDI-TOF MS analysis, they were deemed unidentified. A cut-off score of ≥1.7 was employed as a threshold for quality control. All pure *E. coli* isolate colonies were stored at −80 °C in Brain Heart Infusion (BHI) broth containing 15% glycerol for subsequent investigations.

### 2.3. DNA Extraction from E. coli Isolates

DNA was extracted from a single colony of verified *E. coli* cultured overnight on nutrient agar plates at 37 °C. Each colony was inoculated into 200 μL of sterile distilled water and vortexed for two minutes. Cells were centrifuged (Thermo Fisher Scientific, Waltham, MA, USA) at 13,000 rpm for 10 min. After pipetting 500 µL of distilled water into the Eppendorf tubes and vortexing, the cells were lysed for 15 min at 100 °C in a heat block [21]. A Mini Spin centrifuge (Thermo Fisher Scientific, Waltham, MA, USA) was used to remove the cell debris by centrifuging it for 5 min at 10.000 rpm. Immediately after extraction, the supernatant was used as a PCR template. The *E. coli* ATCC 35218 (Thermo Fisher Scientific, Waltham, MA, USA) was used as a positive control, while the negative control was nuclease-free water.

### 2.4. Molecular Identification of E. coli Using uidA PCR Assay

Singleplex PCR was used to amplify the uidA gene using the reaction conditions outlined in Supplementary Table S1. A total of 30 μL of the PCR reaction mixture consisted of the *E. coli* DNA template (4 μL), 15 μL of Taq 2X Master Mix from New England Biolabs distributed by Inqaba Biotech (Pretoria, South Africa) 1 μM each primer (1 μL), and 9 μL of double-distilled water [22]. 

### 2.5. Detection of Virulence Factors, O Serogroups, and Antibiotic-Resistant Genes

For the purpose of this study, Supplementary Table S1 shows the list of primers and the reaction conditions which were used to detect four *E. coli* O serogroups, namely O157 (*rfbE*), O145 (*wzx*), O113 (*wzy*), and O45 (*wzx1*); virulence genes; H7 flagellar protein (*flicH7*); STEC; Shiga toxin 1 (*stx1*) and Shiga toxin 2 (*Stx2*); and antimicrobial-resistant genes carbapenemases (*KPC*, *VIM*, and *NDM*), lincosamide (*mefA/E)*, sulphonamide (*sulI*), streptomycin (*strA*), tetracycline (*tetB*), and macrolides (*msrA* and *ermA*). A programmable DNA thermo-cycler (Eppendorf FlexCycler 2, Hamburg, Germany) was used in all PCR reactions. The PCR master mix DNA template (4 μL), 15 μL of Taq 2X Master Mix from New England Biolabs distributed by Inqaba Biotech (Pretoria, South Africa), 1 μM of each primer (1 μL), and 9 μL of double-distilled water were mixed. A DNA sample (S4DEC) from Shiga toxin (*Stx*)-producing *E. coli* (isolated from our previous study) was used as a positive control [23].

### 2.6. Phenotypic Antimicrobial Resistance

Antimicrobial susceptibility was assessed using the disc diffusion method on Mueller–Hinton agar (Merck, Darmstadt, Germany). After vaccination, the intervertebral discs were placed on a plate and incubated aerobically at 37 °C for 18–24 h. The results were interpreted according to the M100-ED 31:2021 Performance Standards for Antimicrobial Susceptibility Testing by the Clinical and Laboratory Standards Institute [24]. Eight antibiotics were used in this study, including ampicillin (10 μg), ciprofloxacin (5 μg), erythromycin (15 μg), gentamicin (10 μg), penicillin (10 μg), tetracycline (30 μg), imipenem (10 μg) and meropenem (10 μg), purchased from Thermo Fischer Scientific™ (Thermo Fisher Scientific, Waltham, MA, USA). *E. coli* ATCC 25922 was used as the control for antimicrobial resistance determination. A positive Mueller–Hinton agar plate was interpreted if there was an indentation in the *E. coli* inhibition zone or clove-shaped growth of *E. coli* around the disks [25].

## 3. Results

### 3.1. California Mastitis Test (CMT) and Somatic Cell Counts (SCCs)

Out of the 174 cows sampled, the CMT results showed that 84 (54.3%) cows had an indication of intramammary infection. In addition, only 68 (39.1%) of those cows were positive for subclinical mastitis at a cow level based on the SCC assay.

### 3.2. Identification of E. coli

A total of 68 presumptive *E. coli* strains were isolated from the 174 milk samples collected from dairy cows with subclinical mastitis on six farms. MALDI-TOF-MS analysis and *uidA* gene PCR assay confirmed the identification of all 60 presumptive *E. coli* strains. However, eight isolates were not identified by MALDI-TOF-MS and did not possess the *uidA* gene.

### 3.3. Detection of Virulent Genes and O Serogroups

**Figure 1:**
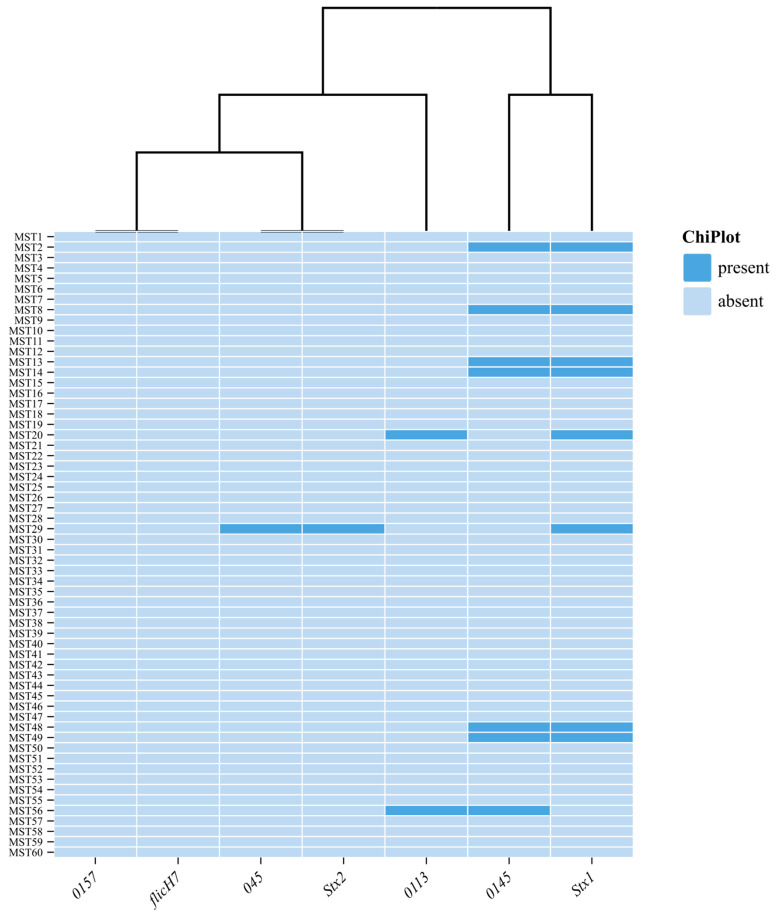
The number of detected virulence genes and serotypes from *E. coli* strains.

The major virulence/toxin genes, including Enterohemorrhagic Flagella H7 *E. coli* (*flicH7*) and *rfbO157,* a virulence gene that codes for the O-antigen specific to the *E. coli* O157:H7 strain and serogroup 0157, were not detected in any of the isolates; nine (15%) isolates harbored *stx1*, and only one isolate harbored *stx2*. One (1.7%) harbored a combination of *stx1* and *stx2* genes. Shiga-like toxin-producing *E. coli* 045 and 0113 were detected in 7/60 (11.7%) and 2/60 (3.3%), respectively (Figure 1).

### 3.4. Antibiotic Sensitivity and Resistance Genes Detected in E. coli Isolates

**Figure 2:**
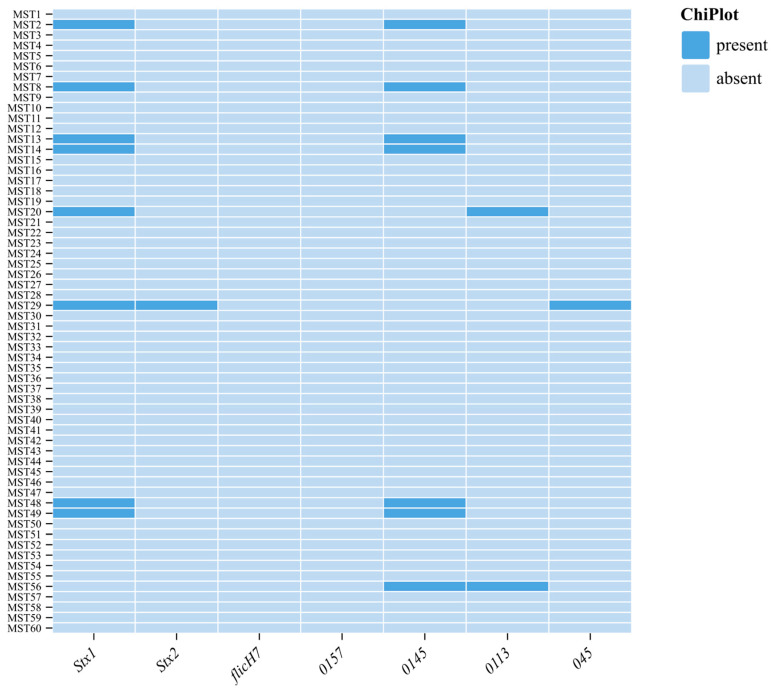
Phenotypic AMR results and their respective AMR genes detected in *E. coli* strains.

According to the results of the disc diffusion test, the majority of the isolates were resistant to penicillin, followed by ciprofloxacin and gentamicin at 43/60 (71.6%), 42/60 (70%), and 18/60 (30%), respectively. Furthermore, 8/60 (13.3%) and 1/60 (1.6%) of the isolates were resistant to meropenem and imipenem, respectively (Figure 2). Genomic antimicrobial resistance profiling indicated that 100% of all isolates carried the *ß*-lactamase gene *bla*_VIM_, followed by *bla*_KPC_ in 23/68 (33.82%) and *bla*_NMD_ in 12/68 (17.64%). Moreover, in 12/68 (12.64%) of the isolates, the *suli1* gene encoding for sulphonamide was also detected, while 1/68 (11.47%) of the isolates were found to have the *msrA* gene encoding for macrolides. Seven (11.6%) isolates were found to harbor both phenotypic and genotypic resistance to carbapenem.

### 3.5. Multidrug-Resistant E. coli Isolates

This study found that 30 out of 60 isolates (50%) resisted three or more antibiotics. Among these isolates, 15 (25%) were resistant to three antibiotics, 12 (20%) were resistant to four, 2 (3.3%) were resistant to six, and 1 (1.7%) was resistant to five antibiotics (Table 1). Of the nine strains of *E. coli* that produce Shiga toxin, only 77.7% were MDR.

## 4. Discussion

Small-scale dairy farming is an important economic sector for improving agriculture and livelihoods in developing nations [26,27]. According to the Food and Agriculture Organization’s 2014 assessment, mastitis is a multifactorial production illness that poses a concern to small-scale producers because of its high prevalence of over 50% [28]. Subclinical mastitis is one of the main issues facing the dairy sector worldwide. The most common cause of subclinical mastitis worldwide is *E. coli* [29].

The current study’s findings about the subclinical mastitis status of cows were consistent with reports of 32.5% and 33% subclinical mastitis, respectively [30]. In contrast to the results of the current study conducted in the same area, subclinical mastitis was found to be 61.60% in the district of Faisalabad’s conventional dairy systems [31]. Likewise, a recent study found that the same district had a 45.9% prevalence of subclinical mastitis [32]. Somatic cell count is a beneficial technique for tracking SCM at individual quarters [33]. The present investigation showed that SCCs increased with higher CMT scores and that the mean SCC was relatively high, ranging from 1.48 to 8.66 × 10^5^ cells/mL of milk. Dos Reis and colleagues concurred that SCCs are the cause of changes in milk supply and composition and increase with CMT scores [34]. Geographical location, breed, age, lactation stages, udder condition, number of parties, immunity, management, hygiene, and milking procedures on dairy farms all affect the incidence of mastitis in lactating cows [35,36].

The present study used PCR targeting the *uidA* gene and MALDI-TOF MS to confirm 60/68 (88.2%) of *E. coli* strains. Our findings were nearly identical to those of Islam et al. [36] in Bangladesh (75%) and Shakya et al. [37] in India (81.1%), who isolated the bacteria from goats, chicken meat, chevon meat, raw milk, and human urine and stool samples. However, studies conducted in South Africa by Ntuli et al. [38] (36%) and China by Liu et al. [39] (34.4%) revealed considerably contrasting outcomes, where their results showed a low prevalence of the isolate in bulk milk and individual milk from dairy herds. The high occurrence of *E. coli* in raw milk and milk products is of concern, as it has been associated with fecal contamination and the consequent risk of enteric pathogens in foodstuffs [40].

STEC is considered to be responsible for causing life-threatening diseases like hemorrhagic colitis. STEC is also considered a significant contributor to kidney failure in children. Hence, consumable milk and milk products contaminated with STEC are serious concerns [41]. The results of the current investigation show that 15% of the isolates harbored *sxt1*, while only one harbored the *stx2* gene encoding for STEC. This is in agreement with the findings of Momtaz et al. [42], Tavakoli and Pourtaghi [43], and Mashak [44], who identified STEC in 13.88%, 22.2%, and 7.7% of the isolates reported from Shahrekord, Tehran, and Alborz Province, respectively, in Iran; the simultaneous presence of *stx1* and *stx2* genes was found in 20.51% of the STEC isolates, in contrast with the current study [42,43,44]. According to Momtaz et al. [42] and Zafarane et al. [45], raw milk from Iranian cows with subclinical mastitis showed a distribution of *stx1* and *stx2*, with 15.06% and 11.11%, respectively. Their findings were supported by those of Brenjchi et al. [46], who found that raw bulk tank milk samples sent to Mashhad’s Pegah Pasteurization Factory exhibited a 6.15% frequency of STEC, with O157:H7 found in just one isolate (12.5%) of these samples.

In contrast, the current study found the prevalence of pathogenic STEC non-0157, which indicates milk contamination and potential public health risks in the dairy value chain system. The prevalence of Shiga-like toxin-producing *E. coli* 045 and 0113 was detected in 11.7% and 3.3%, respectively. This was in agreement with the results of Ullah et al. [47], who found STEC in 5% of bovine milk samples in Pakistan. By comparison, this subgroup was present in Germany at a rate of 24.7%, Egypt at 18.0%, California at 58.1%, and Spain at 35.9%. In this study, serogroup O157 was found to be non-existent, whereas other studies reported low prevalences of 3.8% in Spain and 0% in Egypt. The non-O157 STEC subgroup’s superiority over O157 is therefore consistent with the study conducted by Amézquita-López et al. [48], who reported the prevalence of O157 and non-O157 STEC ranging from 0.42 to 74% and 0.2 to 48.8%, respectively, in Mexico. The variations in prevalence values might be due to the differences in epidemiological determinants like stocking density, age, season, spatial distribution, sampling time, strategy, handling, and laboratory practices.

In the current investigation, the isolates tested positive for the *bla*_KPC_, *bla*_NDM_, and *bla*_VIM_ carbapenemase genes, regardless of whether they were imipenem- or meropenem-resistant. Our study’s findings were consistent with those of another study conducted in Uganda by Okoche et al. [49], which also identified *bla*_VIM_, *bla*_KPC_, and *bla*_NDM_ genes. However, the most common gene was *bla*_VIM_ (10.7%), followed by *bla*_KPC_ (5.1%) and *bla*_NDM-1_ (2.6%). A 2019 study by Hoelle et al. [50] in the USA revealed that roughly 55% of the *E. coli* isolates tested positive for the *bla*_VIM_ gene and 1% for the *bla*_IMP_ gene. These discrepancies may be the result of differences in the samples gathered, the location of the collection, and their large sample sizes. A previous study suggested that beta-lactamases by Gram-negative organisms are usually secreted, especially when antibiotics are present in the environment [51], which probably explains the observation of unexpressed genes in our strain collection.

This study’s results further revealed that most isolates were resistant to penicillin, followed by ciprofloxacin and gentamicin. Our study was similar to those of Zafarane et al. [45] and Tavakoli and Pourtaghi [43], who found that most *E. coli* isolates were resistant to penicillin in Iran. Moreover, a study by Tahar et al. [51] found that ciprofloxacin was also among the antibiotics that *E. coli* was greatly resistant to in Algeria, at 13.5%, even though it was lower than our prevalence. The rate of 18% for gentamicin was comparable to that found in Egypt (13.3%) [52], Tunisia (19.5%) [17], and China (12%) [16]. Furthermore, compared to published rates of 67.9%, 77%, and 49.2% in Brazil [53], Lebanon [54], and Iran [55], the current study’s results were in contrast. In this study, the prevalence of MDR *E. coli* was 30%. This prevalence was higher than the 14.8% recorded by Ngaywa et al. [56] in comparable investigations carried out in Egypt and Northern Kenya, respectively. Nevertheless, it was comparable to the 34.7% noted by Dowidar and Khalifa [57].

## 5. Conclusions

In conclusion, there is a serious risk to animal health, dairy productivity, and public safety due to the high frequency of subclinical mastitis (39.08%) and the predominance of *Escherichia coli*, especially multidrug-resistant strains that carry crucial resistance genes. The zoonotic potential of these pathogens and their function as antimicrobial resistance (AMR) reservoirs in the food production chain were highlighted by the identification of virulence genes (*Stx1* and *Stx2*) and resistance determinants, including *bla*_VIM_, *bla*_KPC_, and *bla*_NDM_. In order to reduce productivity losses and stop the spread of infection, the results call for improved screening programs for the early detection and management of subclinical mastitis in dairy herds. To support evidence-based policy and One Health initiatives, ongoing AMR surveillance in cattle and the environment is crucial, as is genetic tracking of resistance genes.

## Figures and Tables

**Table 1:** Multi-drug resistance *Escherichia coli* isolates.

Antibiotic Disks	Number of Isolates	Percentage
P, CIP, MEM	1	1.6
P, TET, IMP	1	1.6
P, CIP, CN	8	13.3
P, CIP, AMP	3	5
P, CIP, AMP, CN	1	1.6
P, CIP, AMP, E	2	3.3
P, CIP, AMP, TET	4	6.6
P, CIP, E, TET	1	1.6
P, CIP, CN, MEM	1	1.6
P, AMP, MEM, CN	2	3.3
P, AMP, MEM, IMP	1	1.6
E, AMP, CIP	1	1.6
E, CN, CIP, MEM	1	1.6
AMP, CN, CIP	1	1.6
P, CIP, CN, IMP, MEM	1	1.6
P, E, TET, CIP, IMP, MEM	1	1.6

P = Penicillin, CIP = Ciprofloxacin, CN = Gentamicin, MEM = Meropenem, E = Erythromycin, TET = Tetracycline, AMP = Ampicillin.

## Data Availability

All data generated in this study are contained within the manuscript.

## Supplementary Materials

The following supporting information can be downloaded at: https://www.mdpi.com/article/10.3390/ani15131980/s1, Figure S1: The study site where samples were collected; Table S1: The primers used in this study.
