# Supplementary material for: Antimicrobial Resistance and Virulence Determinants of Escherichia coli Isolates from Raw Milk of Dairy Cows with Subclinical Mastitis
Source: Animals (Basel). 2025 Jul 5;15(13):1980. doi: 10.3390/ani15131980 (PMC12248459; doi:10.3390/ani15131980)
Supplement: Supplementary file 1 [file animals-15-01980-s001.zip › animals-3696059-supplementary.pdf]

Supplementary Table S1: Antibiotic resistance and virulence primers used in the study

	Target Gene	Primer sequences (5′-3′)	Product (bp)	Reaction conditions	Reference
Specie-specific	*uidA*	F-CCAAAAGCCAGACAGAGT<br>R-GCACAGCACATCAAAGAG	623	30 cycles (94°C for 4 min, 94, 53, and 72°C for 1 min), and 72°C - 5 min.	[27]
E. coli Virulence genes	*Stx1*	F-AGTTAATGTGGTGGCGAAGG<br>R-CACCAGACAATGTAACCGC	347	35 cycles (94°C for 60 s, 57°C for 60 s, 72°C for 60 sec)	[29]
	Stx2	F-TTCGGTATCCTATTCCCGG<br>R-CGTCATCGTATACACAGGAG	589		
	flicH7	F-ACCATCGGTGGAAGCCAG<br>R-GAAGCATACCCGGCAACAG	461	35 cycles (9 °C for 5 min, followed by 94°C for 30 sec, 63°C for 30 sec, and 72°C for 80 sec)	[30]
O serogroups	*O157*	F-CGGACATCCATGTGATATGG<br>R-TTGCCTATGTACAGCTAATCC	259	30 cycles (95°C for 3 min, 95°C for 20 sec, 58°C for 40 sec, 72°C for 30 sec and 72°C for 8 min.)	[31,32]
	O145	F-CCATCAACAGATTTAGGAGTG<br>R-TTTCTACCGCGAATCTATC	609		
	O113	F-GGGTTAGATGGAGCGCTATTGAGA<br>R-AGGTCACCCTCTGAATTATGGCAG	771	34 cycles (94°C for 6 min, 95°C for 50 sec, 58°C for 70 sec, 72°C for 55 sec and 72°C for 10 min)	[31,32]
	O45	F-CCGGGTTTCGATTTGTGAAGGTTG<br>R-CACAACAGCCACTACTAGGCAGAA	527		
Antibiotic resistance genes	*KPC*	F-CGTCTAGTTCTGCTGTCTTG<br>R-CTTGTCATCCTTGTTAGGCG	789	30 cycles (94°C for 3 min, 95°C for 1 min, 55°C for 31 sec, 73°C for 1 min) and 72°C for 5 min	[33]
	VIM	F-GGTCTCATTGTCCGTGATGGTG<br>R-GGAATCTCGCTCCCCTCTACCT	242	35 cycles (3 min at 94°C, 94°C for 45 sec, at specific annealing temperature (VIM: 60°C and NDM: 52°C) for 45 sec, then at 72°C for 45 sec)	[34]
	NDM	F-GGTTTGGCGATCTGGTTTTC<br>R-CGGAATGGCTCATCACGATC	521		
	mefA/E	F-CGTATTGGGTGCTGTGATTG<br>R-TATGCACAGGCGTTCCATTA	248	35 cycles (95°C for 1 min, 95°C for 30 sec, 60°C for 30 sec, 72°C for 30 sec, and 72°C for 7 min)	[35]
	sulI	F-GACGAGATTGTGCGGTTCTT<br>R-AGGGTTTCCGAGAAGGTGAT	350		
	strA	F-CCGTCAATCCCGACTTCTTA<br>R-CCAGTTCTCTTCGGCGTTAG	263		
	tetB	F-CTCCTTGGCTTGGAAAAATG<br>R-AACCAACCGAACCACTTCAC	229		
	msrA	F-AAGGCTTGTCCGCAATACAC<br>R-CCATTACCCCCAATAAGTGC	320		
	ermA	F-TCAATGGTTGATGTCGTTCA<br>R-AGAAGGGATTTGCGAAAAGA	165		

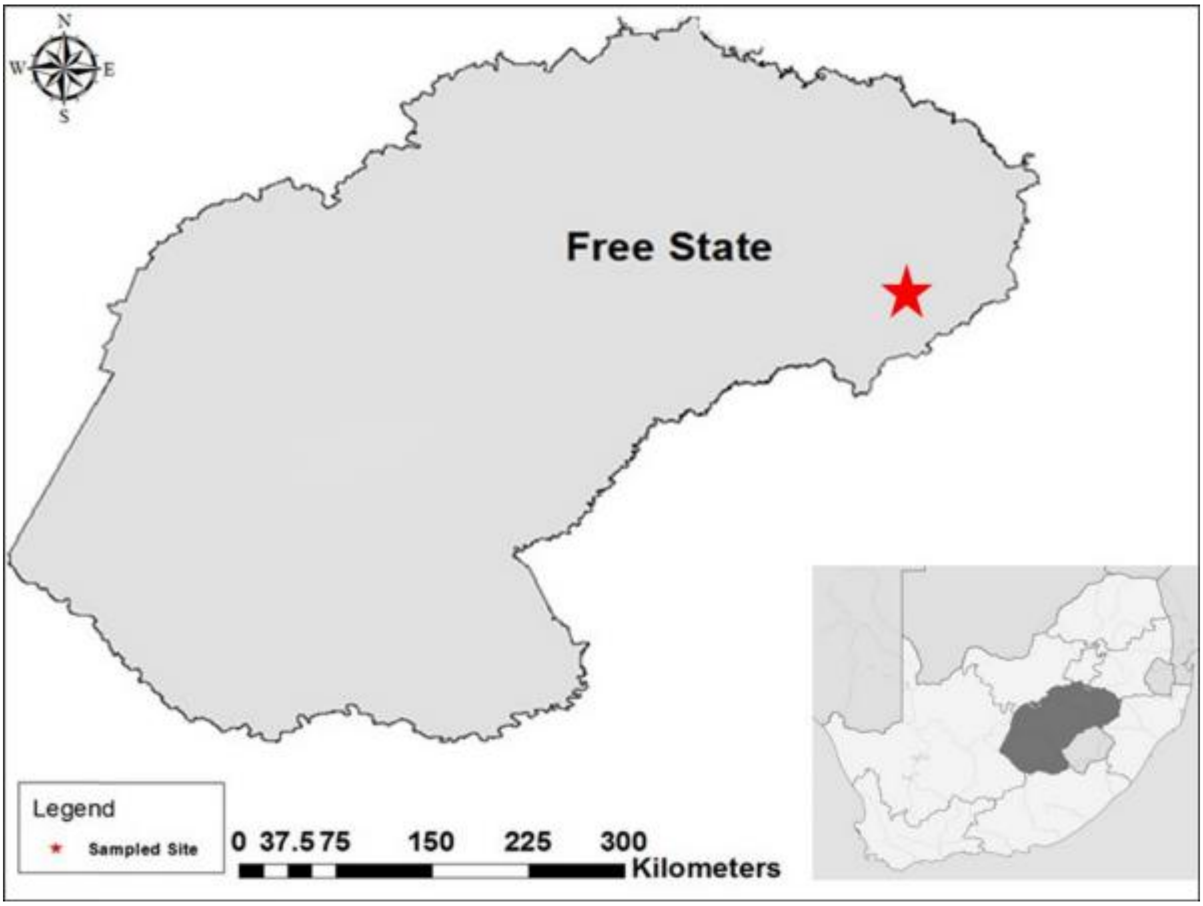

Supplementary Figure S1: Free state province map of South Africa. The star indicates the sampled area.
